# From motorised to active travel: using GPS data to explore potential physical activity gains among adolescents

**DOI:** 10.1186/s12889-022-13947-7

**Published:** 2022-08-09

**Authors:** Venurs Loh, Shannon Sahlqvist, Jenny Veitch, Lukar Thornton, Jo Salmon, Ester Cerin, Jasper Schipperijn, Anna Timperio

**Affiliations:** 1 Institute for Physical Activity and Nutrition (IPAN), School of Exercise and Nutrition Sciences, Deakin University, 221 Burwood Highway, Burwood, Geelong, VIC 3125 Australia; 2 Department of Marketing, Faculty of Business and Economics, University of Antwerp, Antwerp, Belgium; 3 Mary MacKillop Institute for Health Research, Australian Catholic University, Melbourne, Australia; 4 Department of Sports Science and Clinical Biomechanics, University of Southern Denmark, Odense, Denmark

**Keywords:** Youth, Private vehicle, Sustainable travel, Walking, Cycling, Modal shift

## Abstract

**Background:**

A high proportion of adolescents worldwide are not doing enough physical activity for health benefits. Replacing short motorised trips with walking or cycling has the potential to increase physical activity at the population level. This study aimed to estimate the proportion of short distance motorised trips that could be replaced with walking or cycling, and the potential physical activity gains by sociodemographic and trip characteristics.

**Methods:**

Data were from a subsample of the NEighbourhood Activity in Youth (NEArbY) study conducted among adolescents in Melbourne. A total of 217 adolescents with at least one motorised trip completed a survey and wore a Global Positioning Systems (GPS) device for eight consecutive days. Classification of travel modes were based on speed. GPS data points were geocoded in ArcGIS. Motorised trips within walkable (1.3 km) and cyclable (4.2 km) distances were identified (threshold based on 80^th^ percentile of walking and cycling trip distances among Victorian adolescents), and the additional physical activity minutes that could be accrued by replacing walkable or cyclable motorised trip to active trips were quantified. Multilevel linear regression was used to assess differences in physical activity minutes gain by sociodemographic and trip characteristics.

**Results:**

A total of 4,116 motorised trips were made. Of these, 17% were walkable and 61% were cyclable. Replacing motorised trips by walking and cycling resulted in estimated gains of six minutes and 15 min of physical activity per day, respectively.

**Conclusion:**

The sizable proportion of replaceable trips and potential physical activity gains from this shift calls for attention to improve safe and connected infrastructure to support active travel.

## Background

It is well established that regular physical activity during adolescence has significant short- and long-term health benefits [1, 2]. However, fewer than one in five adolescents worldwide [3] and only one in 12 adolescents in Australia [4] achieve the recommendation of 60 min of daily moderate-to-vigorous-intensity physical activity (MVPA). Promotion of physical activity during adolescence is critical as physical inactivity during this life stage is likely to contribute to the epidemic of chronic diseases in adulthood [5, 6].

Active travel, the use of non-motorised transport such as walking or cycling to places, has been recognised as a key strategy to combat the global pandemic of physical inactivity [7]. Evidence from epidemiological research suggests that active travel confers a myriad of health benefits for adolescents, including improved cardiorespiratory fitness [8, 9], reduction of cardiometabolic risk factors [10] and better mental health and wellbeing [11]. Wider benefits of active travel include the development of independent mobility [12], improved navigation skills, and reductions in traffic congestion, carbon footprint and air pollution [13]. In recognition of these benefits, international and national public health agencies (e.g., World Health Organization [14], United Nations[7], National Heart Foundation of Australia [15]) have prioritised the promotion of active travel as a safe, sustainable, enjoyable and equitable mode of travel.

In Australia, car travel is the most dominant mode of travel [16]. More than half (52%) of adolescents in Victoria reported using a private motor vehicle as their main mode of travel to school [17]. Previous studies have observed declines in the use of active travel, either as the main mode of travel or as part of a public transport trip, when adolescents obtain their driver’s license [18–20]. For example, evidence from the US found that obtaining a driver’s license at ages 16–18 years was associated with 40% decline in the number of walking trips [20]. Importantly, studies from the transportation field suggest that car users have the highest probability of ‘sticking’ to their travel mode compared to those who use multiple modes of travel (e.g., combination of walking and public transport) [21, 22]. Therefore, it is important to promote active travel among adolescents before habitual car driving patterns are established.

Car-dependency has been associated with various personal, parental, social, and environmental factors [23]. While not all trips can feasibly be walked or cycled, interventions focusing on shifting short distance motorised trips to walking or cycling may be a promising strategy to improve physical activity at the population level. Several studies among adults in the Netherlands [24, 25] and Australia [26] have examined health and environmental benefits of substituting short distance car trips (up to 7.5 km) with walking and cycling. They found that substituting car trips with walking or cycling decreases economic and disease burden (expressed in Disability-Adjusted Life Years) related to physical inactivity, road traffic noise, and air pollution [25, 26]. These studies, however, used varying definitions of ‘short’ car trips, possibly reflecting contextual differences and none included adolescents.

A recent study based on travel survey data found that 8% of private vehicle trips made by adolescents were replaceable entirely or in part with walking and almost half of private vehicle trips could be replaced entirely or in part with cycling [27]. Trips made for social or shopping reasons and trips made during daylight hours were more likely to be replaceable by active travel than trips to school and trips made during non-daylight hours [27]. That same study also found that total trips, trip purpose and trip modes varied by area-level disadvantage, and that older adolescents (≥ 16 years) and those living in outer-Melbourne had lower odds of having motorised trips that could be replaced with walking or cycling than their counterparts who were younger and those living in inner-Melbourne (region closer to Central Business District). However, that study relied on self-reported trips and did not quantify potential physical activity gains by sociodemographic and trip characteristics. Using Global Positioning Systems (GPS), this study aimed to examine the proportion of short distance motorised trips that could be replaced with walking or cycling, and the potential physical activity gains by sociodemographic and trip characteristics. Understanding swappable motorised trips can inform the development and implementation of tailored strategies to shift adolescents’ travel behaviour from motorised to walking or cycling. Specifically, to inform who should be targeted and tailored messages for trips with particular characteristics.

## Methods

### Participants

Data were collected as part of the NEighbourhood Activity in Youth (NEArbY) study [28]. In brief, data collection occurred from August 2014 to December 2015. For each statistical area level 1 (SA1- the smallest administrative unit for the release of census data used by the Australian Bureau of Statistics [ABS]), median household income and walkability index across Melbourne were calculated and ranked according to four strata: (i) high walkable/high income, (ii) high walkable/low income, (iii) low walkable/high income, and (iv) low walkable/low income.

Secondary schools located in each of these strata were invited to participate, with 18 (of 137) agreeing to participate. Each participating school provided weekly school start and school end times for each school day and facilitated distribution of recruitment packs to interested students. Written parental consent and student assent were received from 528 participants to wear an accelerometer and complete a survey at school. All participants were given the option to also wear a Global Positioning Systems (GPS) device, and consent/assent to wear this additional device was received from 405 participants. Of these, 356 participants received the GPS devices at schools (the remainder were not present or changed their mind). Trained research staff provided instructions on how to wear and position the GPS device on the same belt, as well as how to charge the GPS device at night. Participants were instructed to wear the two devices for eight consecutive days (except during water-based activities and while sleeping). The current sample is based on the subsample who provided GPS data.

### Instruments

#### GPS receiver

Movement between geographical locations over time were recorded using the QStarz BT-Q1000XT GPS receiver. The latitude and longitude of participants’ time-location patterns were collected at 15-s epochs. This GPS receiver has been shown to be highly accurate while stationary [29] and while moving [30].

#### Socio-demographic information

Age (years) and sex were self-reported by the adolescents in the survey. Area-level disadvantage was obtained from the ABS’s Index of Relative Socioeconomic Disadvantage (IRSD) at the SA1 level [31]. The IRSD measure reflects the overall level of disadvantage based on 17 variables that capture a wide range of socioeconomic characteristics, such as education, income, occupation, and household structure. As recommended by the ABS [31], the state-specific (Victoria) IRSD deciles were used for analysis, with the 1^st^ decile denoting the most disadvantaged areas and the 10^th^ decile denoting the least disadvantaged areas.

### Trip identification

Time-stamped GPS data were processed and cleaned using the Personal Activity Location Measurement System (PALMS), a web-based application tool [32]. Extreme speed (> 130 km/h) and changes in elevation (> 1000 m) between two consecutive data points were removed. Using validated trip detection and transport mode classifications [32], PALMS determined whether each data point at every 30 s was part of a trip and classified the trip mode as walking (90^th^ percentile of speed between 1 and < 10 km/h); cycling (90^th^ percentile speed between 10 and < 35 km/h); or motorised (e.g. car, bus, train) (90^th^ percentile speed of ≥ 35 km/h) [33].

A valid trip was defined as a single-way movement from one place to another, covering a distance of at least 100 m and with a duration of at least two minutes. Pauses of up to three minutes in each trip were allowed to account for brief stops, but not at the trip origin or destination. The length, duration, speed and activity level for each trip was identified. Of the 332 files processed by PALMS, a total of 42,703 trips by 323 adolescents were identified.

To address our study aim, motorised trips were extracted for further processing and analyses (*n* = 6,023 trips by 317 adolescents). Motorised trips were classified as originating and ending, respectively, at home, school or ‘elsewhere’. Home and school locations were geocoded. For each participant, trips starting or ending within a 50 m buffer of their home or school polygon were classified as starting or ending at ‘home’ or ‘school’, respectively using ArcGIS Pro version 2.4.3 (ESRI, Redlands, California). A 50 m buffer was used to account for potential inaccuracies of GPS signal and variations in road widths for those being dropped off by vehicle outside the school perimeter. The use of this buffer size is also consistent with previous GPS-based studies among children [34, 35] and adolescents [33]. Trip origin and destination points not within the home and school buffers were coded as ‘elsewhere’. We considered school trips to be those that ended at school (home to school, elsewhere to school, school to school); home trips to be those that ended at home (school to home, elsewhere to home, home to home); and trips to elsewhere to be those that ended elsewhere (home to elsewhere, school to elsewhere, elsewhere to elsewhere). Trips that commenced after sunrise and before sunset were classified as daylight trips; trips that commenced before sunrise or after sunset time were classified as non-daylight trips. Daylight and non-daylight hours were determined using sunset and sunrise time obtained from the National Oceanic Atmospheric Administration’s (NOAA) Solar Calculator algorithm [36], based on the centroid of each participant’s postcode and the date.

For the purpose of this study, we only included participants aged between 12–17 years who had at least one motorised trip. We also only included participants who had worn the GPS device for ≥ 4 h outside school hours for ≥ 3 days on weekdays, and ≥ 8 h for ≥ 1 weekend day to ensure that trip patterns were habitual. Motorised trips within school hours were excluded as these trips were likely to be organised by the school and are non-discretionary.

## Identifying ‘walkable’ and ‘cyclable’ trips

Consistent with a previous study among adolescents in Melbourne [27], we considered ‘walkable’ trips to be ≤ 1.3 km and ‘cyclable’ trips to be ≤ 4.2 km in length. These distance thresholds were determined at the 80^th^ percentile of reported walking and cycling trips among adolescents using the Victorian Integrated Survey of Travel and Activity (VISTA) data [27]. The VISTA is a household travel survey of residents living in Melbourne and Geelong. Any motorised trip within the walkable or cyclable thresholds were considered trips that could potentially be replaced by walking or cycling, respectively.

## Determining additional MVPA accrued

MVPA that could be accrued by shifting travel mode was quantified according to two scenarios: 1) switching motorised trips within walkable distance (≤ 1.3 km) to walking; and 2) switching motorised trips within cyclable distance (≤ 4.2 km) to cycling. According to the youth compendium of physical activities [37], casual self-paced walking and cycling are both equivalent to MVPA. Minutes of MVPA gained from switching to walking (using a casual self-paced walking speed of 4.8 km/h) or cycling (self-paced cycling speed of 16 km/h) [37] according to trip and sociodemographic characteristics were estimated using this formula: ((distance/speed)*60). For example, a 1.3 km motorised trip replaced with walking (average speed: 4.8 km/h) would result in a gain of 16.3 min of MVPA ((1.3/4.8)*60).

### Data analyses

Of the 6,023 motorised trips made by 317 adolescents, 1,907 trips made by 100 adolescents were excluded as follows: non-valid (*n* = 10), made within school hours (*n* = 675), trips of participant that did not meet minimum GPS wear time criteria (*n* = 925), trips made by participant aged 18 and above (*n* = 68) and trips by participant with missing sociodemographic data (*n* = 229). This reduced the analytical sample to 4,116 motorised trips made across 1,268 days by 217 adolescents.

Median distance and duration of trips were calculated due to the skewed data distribution. The proportion of motorised trips that could be replaced by walking and cycling were calculated. The mean MVPA minutes that could be gained by replacing short distance motorised trips with walking or cycling were estimated (the dependent variable). Differences in the additional MVPA minutes gained by replacing motorised trips with walking or cycling by sociodemographic and trip characteristics were estimated using multilevel linear regression models to account for the hierarchical structure and the nesting of trips within days, within individuals and within schools (four-level nested random intercept model). All models were adjusted for age, sex, area-level disadvantage and residual spatial autocorrelation (by including interaction term of the latitude and longitude of trip destination). Data were analysed using Stata SE 17.

## Results

In this sample, the mean age was 14.8 ± 1.5 years and 59% were girls. The proportions of participants were relatively evenly distributed across area-level disadvantage. About three motorised trips were made per day and the median duration and distance for each of these trips was 8 min and 3.3 km, respectively (Table 1).

**Table 1:** Sociodemographic characteristics of the study sample (*n* = 217 adolescents) and descriptive information of motorised trips

*Person-level*	Adolescents with ≥ 1 motorised trip (*n* = 217)
**Age (mean, SD)**	14.8 (1.5)
**Sex (n, %)**	
Male	89 (41.0)
Female	128 (59.0)
**Area-level disadvantage (n, %)**	
Decile 1 (most disadvantaged)	22 (10.1)
Decile 2	21 (9.7)
Decile 3	21 (9.7)
Decile 4	21 (9.7)
Decile 5	18 (8.3)
Decile 6	28 (12.9)
Decile 7	23 (10.6)
Decile 8	29 (13.4)
Decile 9	17 (7.8)
Decile 10 (least disadvantaged)	17 (7.8)
**Recorded days with ≥ 1 motorised trip, mean (SD)**	6.6 (1.7)
***Day-level***	**Days with ≥ 1 motorised trip (*n* = 1268)**
**Number of motorised trips, mean (SD)**	3.2 (1.9)
**Trip duration (mins), median (IQR)**	26 (13–46.5)
**Trip distance (km), median (IQR)**	10 (4.6–22.4)
***Trip-level***	**Motorised trips (*n* = 4116)**
**Trip duration (mins), median (IQR)**	8 (5–13.5)
**Trip distance (km), median (IQR)**	3.3 (1.6–6.6)

### Motorised trips within walkable and cyclable distances

Table 2 presents the proportion of trips within walkable and cyclable thresholds by trip destination and the time of day. Most motorised trips were to elsewhere (70%), 18% were trips to home, and 12% were trips to school. Based on the walkable and cyclable thresholds, 17% of motorised trips were considered walkable and 61% were cyclable. The median distances for motorised trips within walkable and cyclable thresholds were 0.9 km (IQR: 0.6–1.1) and 2.4 km (IQR: 1.7–3.2), respectively. The highest median distances travelled were trips from elsewhere to school (3.9 km) and from school to home (3.9 km). For school trips (*n* = 487), about 14% could be replaced by walking and 60% could be replaced by bike. For home trips, about 17% could be replaced by walking and 63% could be replaced by bike. For trips to elsewhere, 18% could be replaced by walking and 60% could be replaced by bike. About 80% of all motorised trips were made during the daylight hours, and of these, 17% could be replaced by walking and 61% could be replaced by bike. The median distance travelled during daylight and non-daylight hours were relatively similar. The median distance travelled during weekend days was higher than weekdays.

**Table 2:** Median distance (IQR) and proportion of motorised trips within walkable and cyclable thresholds (row percentage) by trip destinations, trip time of day and day of week

**Trip (*n* = 4116)**		**Median distance in km (IQR)**	**Trips within walkable threshold (≤ 1.3 km)** ***n* = 701 (17%)**	**Trips within cyclable threshold (≤ 4.2 km)** ***n* = 2489 (60.5%)**
	**n trips (%)**		**n (row %)**	**n (row %)**
**Trip destinations**				
**School trip**	**487 (11.8)**	**3.4 (1.7, 5.6)**	**69 (14.2)**	**293 (60.2)**
Home to school	229 (5.6)	3.3 (1.6, 6.1)	29 (12.7)	143 (62.5)
Elsewhere to school	237 (5.8)	3.9 (2.0, 5.6)	26 (11.0)	129 (54.4)
School to school	21 (0.5)	0.8 (0.5, 1.4)	14 (66.7)	21 (100)
**Home trip**	**739 (17.9)**	**2.8 (1.5, 6.1)**	**122 (16.5)**	**464 (62.8)**
School to home	72 (1.7)	3.9 (1.6, 6.5)	4 (5.6)	39 (54.2)
Elsewhere to home	590 (14.3)	3.0 (1.6, 6.5)	89 (15.1)	362 (61.4)
Home to home	77 (1.9)	1.4 (0.9, 2.8)	29 (37.7)	63 (81.8)
**Trip to elsewhere**	**2890 (70.2)**	**3.2 (1.7, 6.5)**	**510 (17.6)**	**1732 (60.0)**
Elsewhere to elsewhere	1898 (46.1)	3.4 (1.7, 6.9)	327 (17.2)	1105 (58.2)
Home to elsewhere	704 (17.1)	3.1 (1.4, 6.3)	150 (21.3)	440 (62.5)
School to elsewhere	288 (7.0)	3.0 (1.7, 5.5)	33 (11.5)	187 (64.9)
**Trip time of day**				
Non-daylight	819 (19.9)	3.4 (1.6–6.7)	155 (18.9)	480 (58.6)
Daylight	3297 (80.1)	3.2 (1.6–6.3)	546 (16.6)	2009 (60.9)
**Day of week**				
Weekday	3044 (74.0)	3.0 (1.6–5.9)	533 (17.5)	1920 (63.1)
Weekend day	1072 (26.0)	3.9 (1.7, 7.9)	168 (15.7)	569 (53.1)

### Quantification of MVPA minutes gain from replacing motorised trips with active travel

On average, replacing motorised trips within a walkable distance to walking (scenario 1) would result in estimated gains of 1.8 min of MVPA per trip (95% CI 1.69, 1.96) and 5.9 min of MVPA per day (95% CI 5.43, 6.44). In scenario 2, on average, replacing motorised trips within cyclable distances by bike would result in estimated gains of 4.6 min of MVPA per trip (95% CI 4.47, 4.76) and 15 min of MVPA per day (95% CI 14.28, 15.69).

Differences in MVPA minutes gained from shifting motorised travel to active travel by sociodemographic- and trip characteristics are shown in Table 3. After adjusting for potential confounders, the estimated MVPA gained in scenario 1 per trip was 0.40 min higher for trips made on weekdays compared to weekend days. Estimated gains for scenario 1 did not differ by age, sex, area-level disadvantage, trip destination and trip time of day.

**Table 3:** Associations between estimated gains in MVPA (mins/trip) from shifting motorised trip to active trip according to sociodemographic and trip characteristics

	**Scenario 1**		**Scenario 2**	
	**MVPA gained by switching walkable motorised trips**^**a**^** to walking (mins/trip)**		**MVPA gained by switching walkable or cyclable motorised trip**^**b**^** to cycling (mins/trip)**	
	B (95% CI)	p	B (95% CI)	p
**Age**	-0.07 (-0.20, 0.06)	0.297	-0.01 (-0.19, 0.16)	0.888
Male	Ref		Ref	
Female	0.01 (-0.40, 0.42)	0.963	-0.20 (-0.75, 0.34)	0.464
**Area-level disadvantage**				
Decile 1 (most disadvantaged)	Ref		Ref	
Decile 2	-0.29 (-1.20, 0.61)	0.526	0.48 (-0.73, 1.69)	0.436
Decile 3	-0.38 (-1.25, 0.49)	0.391	-0.32 (-1.50, 0.85)	0.586
Decile 4	-0.53 (-1.43, 0.35)	0.241	0.39 (-0.81, 1.60)	0.517
Decile 5	-0.58 (-1.51, 0.34)	0.216	0.02 (-1.22, 1.27)	0.966
Decile 6	-0.78 (-1.64, 0.08)	0.077	0.28 (-0.87, 1.45)	0.627
Decile 7	0.09 (-0.78, 0.97)	0.830	-0.58 (-1.77, 0.59)	0.332
Decile 8	-0.72 (-1.56, 0.12)	0.096	-0.17 (-1.31, 0.96)	0.763
Decile 9	-0.32 (-1.27, 0.62)	0.508	-0.32 (-1.59, 0.95)	0.625
Decile 10 (least disadvantaged)	-0.26 (-1.19, 0.65)	0.568	-0.27 (-1.53, 0.99)	0.674
**Trip destinations**				
School trip	Ref		Ref	
Home trip	0.18 (-0.29, 0.66)	0.452	-0.22 (-0.75, 0.30)	0.403
Trip to elsewhere	0.24 (-0.16, 0.65)	0.241	-0.21 (-0.35, 1.25)	0.275
**Trip time of day**				
Non-daylight trip	Ref		Ref	
Daylight trip	0.13 (-0.20, 0.47)	0.435	0.48 (0.11, 0.85)	0.010
**Day of week**				
Weekend day	Ref		Ref	
Weekday	0.40 (0.86, 0.72)	0.013	0.88 (0.53, 1.22)	< .0001

Models were adjusted for age, sex, area-level disadvantage and residual spatial autocorrelation

^a^walkable motorised trip is defined as trip distance within 1.3 km

^b^cyclable motorised trip is defined as trip distance within 4.2 km

For scenario 2, the estimated gain per trip was 0.5 min higher for trips made during daylight hours compared to non-daylight hours, and 0.9 min higher for weekday trips compared to weekend trips. There were no differences in the potential minutes of MVPA gained by sociodemographic characteristics and trip destination.

## Discussion

Using GPS data, our findings indicate that a considerable amount of MVPA could be gained by encouraging adolescents to shift short motorised trips to active trips. In particular, replacing short motorised trips by walking and cycling would result in six and 15 min of MVPA gain per day, respectively, contributing up to a quarter of an adolescent’s recommended daily physical activity. We also found that when short distance motorised trips were replaced by walking or cycling, greater minutes of MVPA could be gained for trips made during daylight hours on weekdays than trips made during non-daylight hours on weekend days.

Using the walkable and cyclable thresholds, our study highlights the potential of walking and cycling as a healthy substitute to short distance motorised travel in Melbourne. About one-fifth of motorised trips could be replaced with walking and more than 60% could be replaced with cycling. The proportion of replaceable short distance motorised trips in the current study are higher than our previous findings using the travel survey in Victoria among adolescents (8% of trips could be replaced with walking and 44% could be replaced with cycling) [27]. The discrepancy between the two studies could be due to the over-inflation of short trips in the current study. While the utilisation of GPS receivers allows objective identification of routes to and from places and pauses of up to three minutes were allowed, it is possible that trip segments that were part of the same trip were considered as two separate trips instead of one (i.e., a trip with a brief pause of just over three minutes between the origin and destination may have been considered two trips). The low number of trips from school to home in our study further suggests that participants may have made intermediatory stops before heading home (e.g., trip chaining), or combined motorised travel with other modes (e.g., walked home from a bus stop located > 50 m from home). Nevertheless, the significant proportion of replaceable trips found in both the current study and our previous study [27] highlight the need for supportive infrastructure for active travel, especially infrastructure to support cycling.

When considering distance to destinations, we found that trips to school had the highest median distance compared to trips to home and elsewhere. We also found that walkable and cycleable trips to ‘elsewhere’ were more common than walkable and cycleable trips to school. The importance of non-school trips for physical activity has also been highlighted in other studies. Stewart et al. [33] found that adolescents who do not travel actively to school still accrue a significant proportion of physical activity minutes travelling actively to other places; and Loh et al. [27] found that non-school trips (e.g., shopping, or social trips) were more likely to be replaceable by active travel than school trips. These findings, along with those of the current study, suggest that at least in Australia, interventions to encourage active travel and discourage motorised travel should also focus on non-school journeys as they tend to be shorter and may have less time pressure.

The way we travel has significant impact on population health. The estimated MVPA that we found could be gained from swapping active travel trips is higher than school-based physical activity interventions among children and adolescents, which often have nil to small effects on physical activity [38–40]. For instance, a meta-analysis of school-based interventions on objectively measured MVPA showed a 0.07 increase in MVPA minutes post intervention [38]. Our study also found that greater minutes of MVPA could be gained when short distance motorised trips were replaced by active trips during daylight hours on weekdays than trips made during non-daylight hours on weekend days. Therefore, interventions targeting active trips made during daylight hours on weekdays may further contribute to MVPA minutes gain per day. Despite increased personal exposure to road injuries and environmental hazards such as traffic-related air and noise pollution among active travellers [13, 41], studies have found that the benefits of active travel far outweigh the risk of road- and environmental-related hazards [42–44]. Increasing physical activity through switching short distance trips to active trips is therefore crucial among adolescents who are at risk of low physical activity levels.

### Limitations

There are several limitations that should be acknowledged. It is possible that some travel modes were misclassified as classification was solely based on travel speed. A motorised trip during peak traffic congestion, for example, may have been misclassified as a bicycle trip due to speed. In addition, the trip identification algorithm did not distinguish ‘motorised’ trips and as such there is a lack of specificity as to whether the trip was made by car or public transport. We also did not identify multimodal trips, which may result in overestimated trip numbers and trip destination misclassification. Consistent with previous GPS studies [33, 35], a 50 m buffer was used to classify home and school locations. However, the sensitivity of this distance may have limitations. A 50 m buffer may be too small to capture trips that start or end at a public transport stop near home or school, resulting in data point misclassification as not being a home or school trip, for example. However, using a buffer size larger than 50 m may result in overlap of roads and other destinations [35]. In addition, the use of GPS receivers is subject to positional errors, especially in indoor settings or dense urban areas [45]. The GPS devices were not set to collect signal-to-noise ratio, which may have influenced the identification of the start and end points. The research team attempted to identify the locations of data points that were not within home or school polygons (e.g., shop, recreation facility), but the inter-rater agreement between the researchers was not acceptable (< 60% agreement), and therefore these locations were only reported as ‘elsewhere’. Studies that incorporate tools that provide contextual information (e.g., web-based/smart phone mapping survey, wearable camera) may overcome these limitations and would help to better understand travel behaviours [46, 47].

The estimation of physical activity using the Youth Compendium is unable to account for individual variability due to self-perceived effort, body composition, fitness level and environmental conditions in which the activity is performed. Hence, using the casual self-paced walking and cycling speed reported in the compendium may result in under- or over-estimation of physical activity gains found in the study. While it would have been possible to incorporate physical activity associated with walking trips based on accelerometry in this sample, hip-worn accelerometers do not provide a good estimate of physical activity associated with cycling, even when ‘corrected’ using existing methods [48]. The walkable and cyclable distances were based on the 80^th^ percentile of walking and cycling trips from our previous study [27]. It is important to acknowledge that these distances did not account for other environmental and individual factors that may make walking or cycling less feasible (e.g., hilliness, multimodal trip, carrying heavy goods, lack of skills or confidence to ride a bike). Future studies should explore other barriers and enablers to swapping short distance motorised travel with active travel among adolescents. This study comprised of adolescents living in Melbourne and our findings may not be generalisable to other cities or countries.

## Conclusion

Replacing short motorised trips with active trips can contribute significantly to overall physical activity among adolescents, especially trips made on weekdays during daylight hours. The sizable proportion of motorised trips within cyclable threshold highlights the need for safe and connected infrastructure for cycling to improve environmental and population health. Interventions to promote switching from motorised to active travel should focus on both school and non-school trips during daylight hours on weekdays.

## Data Availability

The dataset used and/or analysed are not publicly available due to ethical restrictions related to the consent given by participants at the time of study commencement. An ethically compliant dataset is, however, available from the corresponding author on reasonable request with approval of the Deakin University Human Research Ethics Committee.

## References

[1] Janssen I, LeBlanc AG (2010). Systematic review of the health benefits of physical activity and fitness in school-aged children and youth. Int J Behav Nutr Phys Act.

[2] Poitras VJ, Gray CE, Borghese MM, Carson V, Chaput J-P, Janssen I (2016). Systematic review of the relationships between objectively measured physical activity and health indicators in school-aged children and youth. Appl Physiol Nutr Metab.

[3] Guthold R, Stevens GA, Riley LM, Bull FC (2020). Global trends in insufficient physical activity among adolescents: a pooled analysis of 298 population-based surveys with 1· 6 million participants. Lancet Child Adolescent Health.

[4] Australian Institute of Health and Welfare (2018). Physical activity across the life stages.

[5] Herman KM, Craig CL, Gauvin L, Katzmarzyk PT (2009). Tracking of obesity and physical activity from childhood to adulthood: the Physical Activity Longitudinal Study. Int J Pediatr Obes.

[6] Abrignani MG, Luca F, Favilli S, Benvenuto M, Rao CM, Di Fusco SA, et al. Lifestyles and cardiovascular prevention in childhood and adolescence. Pediatr Cardiol. 2019;40:1113–25. 10.1007/s00246-019-02152-w 31342115

[7] Environment UN (2016). Global outlook on walking and cycling.

[8] Cooper AR, Wedderkopp N, Wang H, Andersen LB, Froberg K, Page AS (2006). Active travel to school and cardiovascular fitness in Danish children and adolescents. Med Sci Sports Exerc.

[9] Larouche R, Saunders TJ, Faulkner GEJ, Colley R, Tremblay M (2014). Associations between active school transport and physical activity, body composition, and cardiovascular fitness: a systematic review of 68 studies. J Phys Act Health.

[10] Larouche R, Faulkner GE, Fortier M, Tremblay MS (2014). Active transportation and adolescents’ health: the Canadian Health Measures Survey. Am J Prev Med.

[11] Gu J, Chen S-T (2020). Association between active travel to school and depressive symptoms among early adolescents. Children.

[12] Fyhri A, Hjorthol R, Mackett RL, Fotel TN, Kyttä M (2011). Children's active travel and independent mobility in four countries: Development, social contributing trends and measures. Transp Policy.

[13] Cepeda M, Schoufour J, Freak-Poli R, Koolhaas CM, Dhana K, Bramer WM (2017). Levels of ambient air pollution according to mode of transport: a systematic review. The Lancet Public Health.

[14] World Health Organization (2018). Global action plan on physical activity 2018–2030: more active people for a healthier world.

[15] National Heart Foundation of Australia. Blueprint for an active Australia. National Heart Foundation of Australia; 2019.

[16] Australian Bureau of Statistics. More than two in three drive to work, Census reveals 2017. [Available from: https://www.abs.gov.au/ausstats/abs@.nsf/mediareleasesbyreleasedate/7DD5DC715B608612CA2581BF001F8404].

[17] Victorian Integrated Survey of Travel & Activity. Total journeys to education by region and mode 2018 [Available from: https://public.tableau.com/profile/vista#!/vizhome/VISTA-JourneytoeducationAccess/JTE-methodoftravel].

[18] Marique A-F, Dujardin S, Teller J, Reiter S (2013). School commuting: the relationship between energy consumption and urban form. J Transp Geogr.

[19] Chillón P, Martínez-Gómez D, Ortega FB, Pérez-López IJ, Díaz LE, Veses AM (2013). Six-year trend in active commuting to school in Spanish adolescents. Int J Behav Med.

[20] McDonald NC (2006). Exploratory analysis of children's travel patterns. Transp Res Rec.

[21] Janke J, Thigpen CG, Handy S (2021). Examining the effect of life course events on modality type and the moderating influence of life stage. Transportation.

[22] De Haas M, Scheepers C, Harms L, Kroesen M (2018). Travel pattern transitions: applying latent transition analysis within the mobility biographies framework. Transp Res Part A Policy Pract.

[23] Ikeda E, Stewart T, Garrett N, Egli V, Mandic S, Hosking J (2018). Built environment associates of active school travel in New Zealand children and youth: A systematic meta-analysis using individual participant data. J Transp Health.

[24] Scheepers E, Wendel-Vos W, van Kempen E, Panis LI, Maas J, Stipdonk H (2013). Personal and environmental characteristics associated with choice of active transport modes versus car use for different trip purposes of trips up to 7.5 kilometers in The Netherlands. PloS one.

[25] Van Kempen E, Swart W, Wendel-Vos G, Steinberger P, Knol A, Stipdonk H (2010). Exchanging car trips by cycling in the Netherlands: A first estimation of the health benefits. RIVM rapport 630053001.

[26] Zapata-Diomedi B, Knibbs LD, Ware RS, Heesch KC, Tainio M, Woodcock J (2017). A shift from motorised travel to active transport: What are the potential health gains for an Australian city?. PLoS ONE.

[27] Loh V, Sahlqvist S, Veitch J, Carver A, Contardo Ayala AM, Cole R (2022). Substituting passive for active travel—what is the potential among adolescents?. Int J Sustain Transport.

[28] Loh VH, Veitch J, Salmon J, Cerin E, Thornton L, Mavoa S (2019). Built environment and physical activity among adolescents: the moderating effects of neighborhood safety and social support. Int J Behav Nutr Phys Act.

[29] Duncan S, Stewart TI, Oliver M, Mavoa S, MacRae D, Badland HM (2013). Portable global positioning system receivers: static validity and environmental conditions. Am J Prev Med.

[30] Schipperijn J, Kerr J, Duncan S, Madsen T, Klinker C, Troelsen J (2014). Dynamic Accuracy of GPS Receivers for Use in Health Research: A Novel Method to Assess GPS Accuracy in Real-World Settings. Front Public Health..

[31] Australian Bureau of Statistics. Census of Population and Housing: Socio-Economic Indexes for Areas (SEIFA), Australia. Canberra: Australian Bureau of Statistics; 2016. [Available from: https://www.abs.gov.au/ausstats/abs@.nsf/mf/2033.0.55.001].

[32] Carlson JA, Jankowska MM, Meseck K, Godbole S, Natarajan L, Raab F (2015). Validity of PALMS GPS scoring of active and passive travel compared to SenseCam. Med Sci Sports Exerc.

[33] Stewart T, Duncan S, Schipperijn J (2017). Adolescents who engage in active school transport are also more active in other contexts: A space-time investigation. Health Place.

[34] Harrison F, Burgoine T, Corder K, van Sluijs EM, Jones A (2014). How well do modelled routes to school record the environments children are exposed to?: a cross-sectional comparison of GIS-modelled and GPS-measured routes to school. Int J Health Geogr.

[35] Dalton AM, Jones AP, Panter J, Ogilvie D (2015). Are GIS-modelled routes a useful proxy for the actual routes followed by commuters?. J Transp Health.

[36] Cornwall C, Horiuchi A, Lehman C (2017). NOAA solar calculator US Department of Commerce.

[37] Butte NF, Watson KB, Ridley K, Zakeri IF, McMurray RG, Pfeiffer KA (2018). A youth compendium of physical activities: activity codes and metabolic intensities. Med Sci Sports Exerc.

[38] Love R, Adams J, van Sluijs EM (2019). Are school-based physical activity interventions effective and equitable? A meta-analysis of cluster randomized controlled trials with accelerometer-assessed activity. Obes Rev.

[39] Metcalf B, Henley W, Wilkin T (2012). Effectiveness of intervention on physical activity of children: systematic review and meta-analysis of controlled trials with objectively measured outcomes. BMJ.

[40] Neil-Sztramko SE, Caldwell H, Dobbins M. School‐based physical activity programs for promoting physical activity and fitness in children and adolescents aged 6 to 18. Cochrane Database Syst Rev. 2021(9):1-357. 10.1002/14651858.CD007651.pub3 PMC8459921 34555181

[41] Briggs DJ, de Hoogh K, Morris C, Gulliver J (2008). Effects of travel mode on exposures to particulate air pollution. Environ Int.

[42] Mueller N, Rojas-Rueda D, Cole-Hunter T, De Nazelle A, Dons E, Gerike R (2015). Health impact assessment of active transportation: a systematic review. Prev Med.

[43] Maizlish N, Woodcock J, Co S, Ostro B, Fanai A, Fairley D (2013). Health cobenefits and transportation-related reductions in greenhouse gas emissions in the San Francisco Bay area. Am J Public Health.

[44] Rojas-Rueda D, De Nazelle A, Andersen ZJ, Braun-Fahrländer C, Bruha J, Bruhova-Foltynova H (2016). Health impacts of active transportation in Europe. PLoS ONE.

[45] Schipperijn J, Kerr J, Duncan S, Madsen T, Klinker CD, Troelsen J (2014). Dynamic accuracy of GPS receivers for use in health research: a novel method to assess GPS accuracy in real-world settings. Front Public Health.

[46] Chaix B, Kestens Y, Perchoux C, Karusisi N, Merlo J, Labadi K (2012). An interactive mapping tool to assess individual mobility patterns in neighborhood studies. Am J Prev Med.

[47] Chaix B (2020). How daily environments and situations shape behaviors and health: momentary studies of mobile sensing and smartphone survey data. Health Place.

[48] Tarp J, Andersen LB, Østergaard L (2015). Quantification of underestimation of physical activity during cycling to school when using accelerometry. J Phys Act Health.

